# Combined participation in the Supplemental Nutrition Assistance Program (SNAP) and Head Start is associated with healthy household dietary environments for young children in low-income families

**DOI:** 10.1017/S1368980025100864

**Published:** 2025-08-22

**Authors:** Tirna Purkait, Dipti A Dev, Natalie Koziol, Lisa Franzen-Castle, Virginia C Stage, Alison Tovar

**Affiliations:** 1 Department of Nutrition and Health Sciences, University of Nebraska–Lincoln, Lincoln, NE, USA; 2 Department of Child, Youth and Family Studies, University of Nebraska–Lincoln, Lincoln, NE, USA; 3 Nebraska Academy for Methodology, Analytics and Psychometrics (MAP Academy), Nebraska Center for Research on Children, Youth, Families and Schools (CYFS), University of Nebraska–Lincoln, Lincoln, NE, USA; 4 Department of Agriculture and Human Sciences, NC State University, Raleigh, NC, USA; 5 Department of Behavioral and Social Sciences, Brown University, Providence, RI, USA

**Keywords:** Supplemental Nutrition Assistance Program, Head Start, Women, Infants, and Children, Food security, Nutrition security, Nutrition assistance

## Abstract

**Objective:**

To compare the association of participation in Supplemental Nutrition Assistance Program (SNAP) alone *v*. in combination with Head Start (HS), Special Supplemental Nutrition Program for Women, Infants, and Children (WIC) or both on household dietary environment (HDE) indicators: food security, nutrition security, healthfulness choice, dietary choice, perceived food store availability, utilisation barriers and healthy food access barriers in families with young children.

**Design:**

This study, part of SNAP-Ed Nebraska’s Needs and Assets Assessment ‘Healthy People, Healthy State’, utilised a cross-sectional design. HDE indicator means were compared across the federal assistance program (FAP) participation groups using multivariate ANCOVA, controlling for significant demographics, with Benjamini–Hochberg-adjusted *P* values compared with *α* = 0·05.

**Setting:**

Nebraska’s low-income households.

**Participants:**

Households (*n* 821) with at least one child aged 2–6 years participating in SNAP-only (*n* 257), SNAP + HS (*n* 349), SNAP + WIC (*n* 132) and SNAP + WIC + HS (*n* 83).

**Results:**

Compared with other groups, SNAP + HS reported comparatively higher levels of household food security, whereas SNAP + HS + WIC reported lower levels (*P* < 0·01). SNAP + HS also showed higher levels of nutrition security, dietary choices, perceived availability of healthy foods in stores, fewer healthy food access and utilisation barriers (*P* < 0·05).

**Conclusions:**

The findings support recent joint policy changes by Administration for Children and Families and Food and Nutrition Service, facilitating SNAP households’ access to HS. HS performance standards for nutrition and family engagement can serve as a model for creating healthy HDE. Future research should employ quasi-experimental or longitudinal designs to establish causal relationships between FAP participation and HDE outcomes.

One in six households with children across the USA experience food insecurity^(1)^. Children in food-insecure households are more likely to have poor-quality diets, suboptimal nutritional outcomes and decreased cognitive skills due to lower access to healthy food^(2)^. Addressing this critical issue, the U.S. Department of Agriculture (USDA) administers several nutrition assistance programs, targeting the roots of food insecurity and nutritional deficiencies among low-income households with children. Among these, two of the largest federal nutrition assistance programs are the Supplemental Nutrition Assistance Program (SNAP) and the Special Supplemental Nutrition Program for Women, Infants, and Children (WIC)^(3)^. SNAP provides financial assistance to 41·1 million individuals to improve food access, while WIC complements SNAP by supporting 6·3 million mothers, infants and children under 5 with supplemental foods, health care referrals, breast-feeding assistance and nutrition education.^(4)^. In parallel, Head Start (HS), another federal program, supports 833 000 preschool children from low-income families with education, nutrition and health services through childcare centres, family homes and family childcare homes^(5)^. Collectively, SNAP, WIC and HS as federal assistance programs (FAP) can offer coordinated resources that help reduce food insecurity and improve nutrition, thereby laying a foundation for healthier families with young children. A healthy household dietary environment (HDE) requires adequate access to affordable, nutritious foods, which is critical for shaping young children’s dietary patterns and the overall well-being of families, with FAP playing a key role in supporting these dietary environments^(6)^. While individual participation in each of these programmes has been linked to improvements in HDE-related outcomes, few studies have explored how simultaneous participation in these programmes is associated with those outcomes, with some focusing on the joint effects of SNAP and WIC^(7,8)^. The current study aims to bridge this gap in the literature by examining the associations between participation in SNAP alone and in combination with WIC, HS or both with HDE indicators (e.g. food and nutrition security, healthfulness choice, dietary choice, perceived availability, utilisation barriers and healthy food access barriers) in families with 2–6-year-old children. The findings are crucial for resource allocations and developing targeted federal policies and programs.

## Conceptualising household dietary environment

A healthy HDE should is characterised by having consistent and sufficient availability of affordable, healthy foods within the household, including a supply of high-quality products that meet the dietary needs of all family members, and access to local food sources that cater to both families and children’s preferences^(9)^. Assessing both the quantity (food security) and quality (nutrition security) of food access can identify households at risk of diet-related health risks^(10)^. Dietary consumptions are shaped not only by nutritional needs but also by personal preferences (such as taste, flavour and texture), early dietary exposures (like breast-feeding or the timing of solid food introduction), health considerations (including the need to consume a variety of vegetables or follow specific diets like low-Na or high fibre), cultural influences (such as traditional cuisines or religious dietary practices) and the availability of healthy food options (for example, access to fresh produce or whole grains in local stores)^(11)^. Additionally, geographic and economic constraints can limit healthy food access, while barriers to utilisation such as limited culinary knowledge and lack of essential cooking equipment or storage facilities may further add layers to such complexity^(12,13)^. Therefore, HDE is not merely about physical presence of food but involves a nuanced interplay between what is nutritionally needed, personally preferred and practically accessible.

The current study measures the HDE indicators with households’ food security (access to adequate food), nutrition security (perceived ability to acquire healthful foods), healthfulness choice (ability to access preferred healthy foods) dietary choice (ability to accommodate individual food preferences and having control over the available food options), as well as perceived limited food store availability (available healthy options in local stores), utilisation barriers (using food for preparing healthful meals) and healthy food access barriers,^(11–13)^ and provides a comprehensive approach for deeper understanding of the multifaceted factors that collectively influence the dietary environment of households with young children.

The theoretically grounded HDE construct is conceptually rooted in our previously validated Early Care and Education food environment framework, was adapted and operationalised for the household context in this study. The original Early Care and Education framework defined institutional food environments through five validated dimensions: availability, accessibility, affordability, acceptability and accommodation^(9)^. These dimensions informed our adaptation of the HDE construct and also guided the selection of valid and reliable household-level measures described earlier, ensuring theoretical rigor, conceptual clarity and methodological coherence.

## Methods

### Study design, participants and setting

This cross-sectional study, part of SNAP-Ed Nebraska’s needs and assets assessment project (Healthy People, Healthy State), utilised a self-administered 202-item survey developed in Qualtrics. Data collection took place from May 2023 to December 2023. A priori power analysis, utilising values from Friedman^(14)^ and Cohen^(15)^, with some interpolation to minimise the likelihood of type II error, determined that a minimum sample size of *n* 82 in each group (SNAP-only, SNAP + HS, SNAP + WIC, SNAP + HS + WIC) was needed to detect a small effect size (*d* = 0·3) with 80 % power at a significance level of 0·05, to ensure adequate power for group comparisons across HDE indicators. Recruitment across Nebraska was conducted with the help of Nebraska Extension and collaborative partners (e.g. Nebraska Department of Health and Human Services, Nebraska Food Security Task Force). The recruitment flyer included a QR code and Qualtrics survey link available in English, Spanish and Arabic to accommodate diverse language preferences, distributed across urban, rural and food desert areas. Appropriate security measures were implemented to prevent bot responses, including enabling CAPTCHA, incorporating honeypot fields, restricting survey access to one response per device and disseminating the survey through known listservs. Incentives were provided to encourage broad participation, offering $50 Amazon gift card to every thirtieth participant through a randomised raffle.

Eligibility criteria for this study were (i) age ≥ 19 years (Nebraska’s age of majority), (ii) Nebraska residency, (iii) SNAP participation and (iv) families with at least one child aged 2–6 years. Additionally, participants were required to be either at risk for (v) food insufficiency (based on household food situation in the past 7 d, ranging from having enough desired foods to often not having enough to eat) (16) or (vi) food insecurity (based on household food situation over the past 12 months, including concerns about running out of food or not having enough money to purchase more)^(16)^.

Study-eligible respondents (*n* 821) were segmented based on current FAP participation: households exclusively participating in SNAP-only (*n* 257), both SNAP and HS (*n* 349), SNAP and WIC (*n* 132) and combination of SNAP, WIC and HS (*n* 83). The study explores the association of different combinations of SNAP with other two FAP (HS and WIC) on HDE outcomes and hence SNAP-only group served as a common baseline. All households in the analytic sample are SNAP participants with at least one child aged 2–6 years. We included children up to age 6 as some may have just aged out at 5 during data collection period. Children under 2 were excluded as their nutritional needs are primarily met through breast-feeding or formula, making the HDE indicator less relevant for them. SNAP participants are automatically considered income eligible for WIC, and all SNAP households can demonstrate their eligibility for HS. Therefore, all FAP groups in this study were fully eligible for every programme they participate. We refer to both early HS (for children up to 3-years) and HS (for 3–5- year-old children), collectively as HS in this study. We also ensured that no children in our sample reported receiving free and reduced-price school meals to ensure that the outcomes are not influenced by overlapping school nutrition program participation.

The survey was reviewed and adjusted to a grade-5 reading level for low-income, potentially rural participants by an interdisciplinary expert advisory committee and UNL Bureau of Sociological Research. To validate the survey, cognitive interviews with verbal probing^(17)^ were conducted with three eligible participants. No adaptations were made to the survey instruments used in this current research.

Participants’ socio-demographic data (Table 1) included their ZIP codes of residence to classify counties as urban (≥ 50 000 residents) or rural (< 50 000 residents) using rural–urban continuum codes^(18)^. Food deserts were identified based on USDA criteria, where residents live over a mile from a supermarket with incomes below 80 % of the metropolitan median or 20 % below the federal poverty level^(19)^.

**Table 1.** Demographic characteristics across FAP participation groups (*n* 821)

Characteristics	SNAP-only	SNAP + HS	SNAP + WIC	SNAP + WIC + HS	
	(*n* 257)	(*n* 349)	(*n* 132)	(*n* 83)	χ^2^/F^†^
Sex of the respondent (%)					57·49***
Male	31·90	14·60	29·50	51·80	
Female	68·10	85·40	70·50	48·20	
Race (%)					103·986***
White	81·70	94·60	65·20	72·30	
Black/African American	14·80	4·0	23·50	9·60	
American Indian/Alaskan Native	0·80	0·90	2·30	2·40	
Asian	1·20	0·60	7·60	13·30	
Native Hawaiian/ Pacific Islander	1·60	0	1·50	2·40	
Ethnicity (%)					330·067***
Hispanic	16·00	87·10	33·30	38·60	
Non-Hispanic	84·00	12·90	66·70	61·40	
Annual household income (%)					16·691***
$24 999 or less	8·20	2·00	5·40	11·00	
$25 000 or more	91·80	98·0	94·6	89·00	
Education level of the respondent (%)					74·341***
No diploma	4·30	0	5·30	0	
High School Diploma/GED	17·10	6·60	14·40	21·70	
Some college, but no degree	25·70	33·20	19·70	28·90	
Technical/Associate/Junior College	28·40	25·80	18·20	7·20	
Bachelor’s degree	22·60	32·70	38·60	39·80	
Graduate degree	1·90	1·70	3·80	2·40	
Place of birth of the respondent (%)					12·503**
United States	97·70	99·1	93·9	98·8	
Other country	2·30	0·90	6·10	1·20	
Number of family members					
Mean	3·84	5·44	3·76	4·01	139·06***
sd	0·78	1·31	1·05	1·07	
Urban rural status (%)					12·816**
Urban	87·50	76·50	79·50	75·90	
Rural	12·50	23·50	20·50	24·10	
Food desert status (%)					12·153**
Non-food desert	90·70	89·10	97·70	96·40	
Food desert	9·30	10·90	2·30	3·60	

FAP, federal assistance program; SNAP, HS, Head Start; WIS, Women, Infants, and Children; HDE, household dietary environment.

Values are percentages unless otherwise indicated. Due to rounding, totals may not sum to 100 %.

† Comparisons of study groups were made with Pearson’s χ^2^ test (categorical variables) and ANOVA (continuous variables).

*P < 0·05, **P < 0·01, ***P < 0·001.

### Variables

The current study measures the HDE indicator variable using seven instruments with validity and reliability evidence. The Cronbach’s alpha values, ranging from 0·63 to 0·80, span from acceptable to good^(20)^. Psychometric evaluations were conducted using R version 4.2.2 (R Core Team, Vienna, Austria), selected for its advanced capabilities in psychometric analysis and model fit assessments. See online supplementary material, Supplemental Material 1 for all the survey instruments used in this study.

#### Household food security

To measure food security, we employed the 18-item USDA Household Food Security Survey Module^(21,22)^. Cronbach’s alpha was 0·69. As higher raw scores indicate greater severity of food insecurity, we referred this variable as ‘Food Insecurity’ in our HDE outcome results. Although Household Food Security Survey Module typically categorises raw scores into ‘High’ (0), ‘Marginal’ (1–2), ‘Low’ (3–7) and ‘Very Low’ (8–18) food security, we used raw scores to capture variations in food insecurity severity across FAP participation groups^(22,23)^.

#### Household nutrition security, choice, availability and utilisation

Measures developed by the Center for Nutrition and Health Impact (formerly Gretchen Swanson Center for Nutrition) were employed to assess household nutrition security^(24)^, healthfulness choice^(24)^, dietary choice^(24)^, perceived limited availability^(25)^ and utilisation barriers^(25)^. According to Center for Nutrition and Health Impact, higher scores on nutrition security, healthfulness choice, dietary choice measures indicated better outcomes suggesting greater nutrition security, greater healthfulness choice and greater dietary choice^(11,24)^. In contrast, higher scores on perceived limited availability, and utilisation barriers measures indicated more challenges suggesting greater perceived limited availability and more significant utilisation barriers^(12,25)^. Prior research with a racially and ethnically diverse sample of low-income, food-insecure households established that scores of 2·00 or below correspond to ‘low’ levels of nutrition security and related indicators, while scores of 3·00 on perceived limited availability and utilisation barriers scales reflect higher level of challenges^(24,25)^. It is important to note that as per Center for Nutrition and Health Impact guidelines, these indicators are treated as continuous variables, with numeric values representing relative levels on each construct within each FAP participation group. Cronbach’s alpha was 0·71 for nutrition security (4 items), 0·63 for healthfulness choice (three items), 0·68 for dietary choice (three items), 0·76 for food store perceived limited availability (three items) and 0·80 for utilisation barriers (eight items).

#### Healthy food access barriers

Seven key healthy food access barriers were identified from the literature: distance to the grocery store, transportation, limited store hours, cost, physical disabilities, food quality and time constraints^(26,27)^. Responses were scored on a five-point scale from ‘Never’ to ‘Always’ and aggregated into a composite mean score, reflecting the overall severity of food access barriers experienced by respondents. The following tests were conducted to psychometrically evaluate the healthy food access barriers measure, ensuring internal consistency, reliability and construct validity:

##### Bottom of form

Reliability analysis. The first step was to conduct a reliability analysis. Cronbach’s alpha for the 7 items was 0·7, indicating an acceptable level of internal consistency.

Confirmatory factor analysis for model fit. To evaluate construct validity evidence and the internal structure of the measure, confirmatory factor analysis was performed to test whether a single-factor model composed of the seven healthy food access barrier items adequately fit the data. Several fit indices suggested excellent model fit. The comparative fit index and Tucker–Lewis index were both close to 1, comparative fit index = 0·979 and Tucker–Lewis index = 0·968, suggesting very good fit. Additionally, the root mean square error of approximation was 0·035, and the standardised root mean square residual was 0·024, both well within the acceptable range (root mean square error of approximation < 0·06 and standardised root mean square residual < 0·08). These results support the proposed 1-factor structure and provide evidence of construct validity, thereby justifying the use of the composite score as a measure of barriers to healthy food access in our study.

### Statistical analysis

Demographic characteristics and FAP participation data were analysed across four groups: SNAP-only, SNAP + HS, SNAP + WIC and SNAP + HS + WIC. Variables such as respondent’s sex, race, ethnicity, income, education level, place of birth, family size and urban–rural and food desert status were examined for significant differences using Pearson’s χ2 for categorical variables and ANOVA for continuous variables. These demographics were commonly identified as potential confounders associated with HDE indicators^(3,11,12)^. Pearson’s correlation was estimated among these indicators to check for multicollinearity. Multivariate ANCOVA was used to simultaneously assess the equality of means across various HDE indicators among the four FAP participation groups. The SNAP-only group served as reference group. All demographic variables were significantly different across the study groups and therefore included in the MANCOVAs as covariates. *Post hoc* pairwise comparisons were performed for significant omnibus associations. The statistical analyses in this stage were conducted using IBM SPSS Statistics version 29.0.2.0 (IBM Corp.). To control the false discovery rate due to multiple comparisons and maintain a balance between error control and statistical power, the Benjamini-Hochberg correction was applied. This correction was applied in two ways. First, it was used for the omnibus F-tests, with the *P* values adjusted for seven comparisons. Second, it was used for the *post hoc* pairwise comparisons within each outcome measure, with the *P* values adjusted for six comparisons. The significance level for all tests was set at *α* = 0·05, taking into consideration the adjusted *P* values obtained after applying the Benjamini–Hochberg procedure. This methodology allowed for a rigorous and controlled analysis of the data while balancing the risk of Type I and Type II errors. As measures of effect size, adjusted R-squared and partial eta-squared were calculated to assess the proportion of variability in each HDE indicator associated with all variables in the model and uniquely associated with FAP participation group, respectively. Cohen’s *d* and B-H-adjusted 95 % confidence intervals were calculated as a measure of the standardised mean difference in HDE indicators between FAP participation groups.

## Results

This study included *n* 821 respondents from Nebraska families with low-income participating in SNAP-only or in combination with HS, WIC or both. Of these, 31·30 % were enrolled in SNAP-only (*n* 257), 42·50 % in SNAP + HS (*n* 349), 16·08 % in SNAP + WIC (*n* 132) and 10·11 % in SNAP + WIC + HS (*n* 83). The demographic characteristics (Table 1) were examined and revealed significant differences among FAP participation groups. Majority of the respondents were White (83·6 %). The SNAP + HS group had the highest percentage of Hispanic participants (87·1 %), whereas the SNAP + WIC + HS group had the highest percentage of participants with a bachelor’s degree (39·8 %) as well as the highest rural residency (24·1 %).

Descriptive statistics and multivariate ANCOVA-adjusted marginal mean and standard errors for the key measures of HDE, operationalised as a multivariate outcome composed of seven indicators, disaggregated by FAP participation groups, are presented in Table 2. Controlling for the demographic variables, results of the comparative analysis revealed a significant multivariate association between FAP participation and the combined set of HDE indicators (Wilk’s lamda = 0·87, F (21, 2294·85) = 5·26, *P* < 0·001, partial η^2^ = 0·04) and significant univariate associations between FAP participation and each of the HDE indicator (B-H *P* < 0·001 to 0·045; Table 3). The total percentage of variability in HDE indicators associated with all variables in the model ranged from adjusted R^2^ = 16 % (utilisation barriers) to 56 % (food store perceived limited variability), and the unique percentage of variability associated with program participation ranged from partial η^2^ = 1 % (healthfulness choice) to 6 % (healthy food access barriers).

**Table 2.** Descriptive and MANCOVA-Adjusted HDE indicators across FAP participation groups

Dependent variable	Statistic^*^	SNAP-only	SNAP + HS	SNAP + WIC	SNAP + WIC + HS
Food insecurity	M_unadj_	8·99	4·28	8·41	9·83
	sd	4·52	4·65	4·35	5·04
	M_adj_	7·19	6·23	7·67	8·38
	se	0·30	0·28	0·37	0·46
Nutrition security	M_unadj_	2·02	2·78	1·98	2·01
	sd	0·62	0·63	0·77	0·84
	M_adj_	2·31	2·49	2·10	2·15
	se	0·05	0·05	0·06	0·07
Healthfulness choice	M_unadj_	2·01	1·26	1·90	1·97
	sd	0·64	0·69	0·92	0·81
	M_adj_	1·74	1·56	1·74	1·82
	se	0·05	0·05	0·06	0·08
Dietary choice	M_unadj_	1·86	2·80	1·85	1·95
	sd	0·65	0·71	0·79	0·85
	M_adj_	2·14	2·52	1·99	2·07
	se	0·05	0·05	0·06	0·08
Utilisation barriers	M_unadj_	4·93	4·57	5·79	5·28
	sd	2·06	1·79	2·35	2·58
	M_adj_	4·85	4·88	5·47	4·76
	se	0·15	0·14	0·18	0·23
Food store perceived limited availability	M_unadj_	2·41	1·02	2·52	2·43
	sd	0·68	1·07	0·80	0·89
	M_adj_	1·95	1·57	2·24	2·00
	se	0·06	0·05	0·07	0·09
Healthy food access	M_unadj_	2·05	1·24	2·11	2·01
	sd	0·49	0·55	0·58	0·64
Barriers	M_adj_	1·77	1·54	1·98	1·87
	se	0·04	0·04	0·05	0·06

MANCOVA, multivariate ANCOVA; FAP, federal assistance program; HDE, household dietary environment; SNAP, Supplemental Nutrition Assistance Program; HS, Head Start.

* M_unadj_ (sd) = raw mean and SD. M_adj_ (se) = estimated marginal mean adjusted for covariates and se.

**Table 3.** Univariate associations of FAP participation groups on HDE indicators

Dependent variable	Corrected model adjusted R^2^	Omnibus association of FAP participation			
		F (3, 805)	Unadj. *P* ^*^	B-H *P* ^†^	Partial eta-squared (η^2^)
Food insecurity	0·42	5·38	0·001	0·002	0·02
Nutrition security	0·34	9·45	< 0·001	< 0·001	0·03
Healthfulness choice	0·30	2·70	0·045	0·045	0·01
Dietary choice	0·37	13·94	< 0·001	< 0·001	0·05
Utilisation barriers	0·16	3·60	0·013	0·016	0·01
Food store perceived limited availability	0·56	15·63	< 0·001	< 0·001	0·06
Healthy food access barriers	0·48	18·02	< 0·001	< 0·001	0·06

FAP, federal assistance program; HDE, household dietary environment.

* Unadj. *P* = Unadjusted *P* value.

† B-H *P* = Benjamini–Hochberg adjusted *P*-value.

*Post hoc* comparisons revealed significant pairwise differences between FAP participation groups on individual HDE indicators, with associations ranging from small to large (Table 4). |*d*| values are used to show the magnitude of differences between FAP participation groups on HDE indicators, with values interpreted as small (|*d*| ≈ 0·2), medium (|*d|* ≈ 0·5) or large (|*d*| ≈ 0·8) effects^(15)^. On average, SNAP + HS households reported significantly lower food insecurity than SNAP + WIC (|*d*| = 0·32, 95 % CI (–0·56, –0·07)) and SNAP + WIC + HS (|*d*| = 0·45, 95 % CI (–0·78, –0·13)) and higher nutrition security than SNAP-only (|*d*| = 0·29, 95 % CI (–0·47, –0·12)), SNAP + WIC (|*d*| = 0·59, 95 % CI (0·31, 0·86)) and SNAP + WIC + HS (|*d*| = 0·51, 95 % CI (0·21, 0·80)). SNAP + HS participants also showed significantly better dietary choice than SNAP-only (|*d*| = 0·56, 95 % CI (–0·76, –0·36)), SNAP + WIC (|*d*| = 0·73, 95 % CI (0·45, 1·01)) and SNAP + WIC + HS (|*d*| = 0·61, 95 % CI (0·33, 0·88)) and better perceived availability of foods in stores than SNAP-only (|*d*| = 0·41, 95 % CI (0·21, 0·61)), SNAP + WIC (|*d*| = 0·66, 95 % CI (–0·94, –0·38)) and SNAP + WIC + HS (|*d*| = 0·41, 95 % CI (–0·68, –0·13)). SNAP + HS participants reported fewer utilisation barriers than SNAP + WIC (|*d*| = 0·31, 95 % CI (–0·54, –0·08)) and fewer food access barriers than SNAP-only (|*d*| = 0·45, 95 % CI (0·26, 0·64)), SNAP + WIC (|*d*| = 0·79, 95 % CI (–1·07, –0·52)) and SNAP + WIC + HS (|*d*| = 0·59, 95 % CI (–0·88, –0·29)). SNAP + WIC participants reported lower nutrition security than SNAP-only (|*d*| = 0·31, 95 % CI (0·07, 0·55)) and lower perceived food availability than SNAP-only (|*d*| = 0·39, 95 % CI (–0·62, –0·16)) and SNAP + WIC + HS (|*d*| = 0·29, 95 % CI (0·00, 0·57)), as well as greater utilisation barriers than SNAP-only (|*d*| = 0·29, 95 % CI (–0·57, –0·01)) and SNAP + WIC + HS (|*d*| = 0·29, 95 % CI (–0·04, 0·63)). There were no significant pairwise group differences in healthfulness choice.

**Table 4.** *Post hoc* pairwise comparisons between FAP participation groups on HDE indicators

Dependent variable	Group I	Group J	M_Diff_^*^ (I-J)	se ^†^	Unadj. *P* ^‡^	B-H *P* ^§^	*d* ^||^	B-H 95 % CI for *d* ^¶^
Food insecurity	SNAP-only	SNAP + HS	0·95	0·48	0·046	0·069	0·21	0·03, 0·38
		SNAP + WIC	−0·49	0·45	0·275	0·275	−0·11	−0·32, 0·10
		SNAP + WIC + HS	−1·19	0·53	0·024	0·048	−0·26	−0·54, 0·03
	SNAP + HS	SNAP + WIC	−1·44	0·50	0·004	0·013	−0·32	−0·56, –0·07
		SNAP + WIC + HS	−2·15	0·57	< 0·001	0·001	−0·45	−0·78, –0·13
	SNAP + WIC	SNAP + WIC + HS	−0·70	0·57	0·216	0·259	−0·15	−0·44, 0·13
Nutrition security	SNAP-only	SNAP + HS	−0·18	0·08	0·017	0·026	−0·29	−0·47, –0·12
		SNAP + WIC	0·21	0·07	0·003	0·007	0·31	0·07, 0·55
		SNAP + WIC + HS	0·16	0·08	0·060	0·072	0·23	−0·03, 0·49
	SNAP + HS	SNAP + WIC	0·39	0·08	< 0·001	< 0·001	0·59	0·31, 0·86
		SNAP + WIC + HS	0·34	0·09	< 0·001	0·001	0·51	0·21, 0·80
	SNAP + WIC	SNAP + WIC + HS	−0·05	0·09	0·571	0·571	−0·06	−0·34, 0·21
Healthfulness choice	SNAP-only	SNAP + HS	0·18	0·08	0·034	0·101	0·26	0·07, 0·46
		SNAP + WIC	0·00	0·08	0·963	0·963	−0·01	−0·21, 0·21
		SNAP + WIC + HS	−0·08	0·09	0·363	0·544	−0·12	−0·39, 0·15
	SNAP + HS	SNAP + WIC	−0·18	0·09	0·039	0·079	−0·24	−0·47, –0·01
		SNAP + WIC + HS	−0·26	0·10	0·009	0·055	−0·36	−0·69, –0·04
	SNAP + WIC	SNAP + WIC + HS	−0·08	0·10	0·419	0·503	−0·09	−0·38, 0·20
Dietary choice	SNAP-only	SNAP + HS	−0·38	0·08	< 0·001	< 0·001	−0·56	−0·76, –0·36
		SNAP + WIC	0·15	0·08	0·050	0·075	0·22	−0·01, 0·45
		SNAP + WIC + HS	0·07	0·09	0·479	0·479	0·09	−0·16, 0·34
	SNAP + HS	SNAP + WIC	0·53	0·09	< 0·001	< 0·001	0·73	0·45, 1·01
		SNAP + WIC + HS	0·45	0·10	< 0·001	< 0·001	0·61	0·33, 0·88
	SNAP + WIC	SNAP + WIC + HS	−0·09	0·10	0·375	0·450	−0·11	−0·39, 0·18
Utilisation barriers	SNAP-only	SNAP + HS	−0·03	0·23	0·902	0·902	−0·02	−0·18, 0·15
		SNAP + WIC	−0·63	0·22	0·004	0·025	−0·29	−0·57, –0·01
		SNAP + WIC + HS	0·09	0·26	0·735	0·882	0·04	−0·22, 0·30
	SNAP + HS	SNAP + WIC	−0·60	0·25	0·015	0·030	−0·31	−0·54, –0·08
		SNAP + WIC + HS	0·12	0·28	0·679	> 0·999	0·06	−0·20, 0·32
	SNAP + WIC	SNAP + WIC + HS	0·72	0·28	0·010	0·031	0·29	−0·04, 0·63
Food store perceived limited availability	SNAP-only	SNAP + HS	0·38	0·09	< 0·001	< 0·001	0·41	0·21, 0·61
		SNAP + WIC	−0·28	0·09	0·001	0·002	−0·39	−0·62, –0·16
		SNAP + WIC + HS	−0·04	0·10	0·675	0·675	−0·06	−0·31, 0·19
	SNAP + HS	SNAP + WIC	−0·66	0·10	< 0·001	< 0·001	−0·66	−0·94, –0·38
		SNAP + WIC + HS	−0·42	0·11	< 0·001	< 0·001	−0·41	−0·68, –0·13
	SNAP + WIC	SNAP + WIC + HS	0·24	0·11	0·030	0·036	0·29	0·00, 0·57
Healthy food access barriers	SNAP-only	SNAP + HS	0·24	0·06	< 0·001	< 0·001	0·45	0·26, 0·64
		SNAP + WIC	−0·21	0·06	< 0·001	< 0·001	−0·40	−0·63, –0·17
		SNAP + WIC + HS	−0·10	0·07	0·136	0·136	−0·19	−0·43, 0·06
	SNAP + HS	SNAP + WIC	−0·45	0·06	< 0·001	< 0·001	−0·79	−1·07, –0·52
		SNAP + WIC + HS	−0·33	0·07	< 0·001	< 0·001	−0·59	−0·88, –0·29
	SNAP + WIC	SNAP + WIC + HS	0·11	0·07	0·116	0·139	0·18	−0·10, 0·47

FAP, federal assistance program; HDE, household dietary environment; SNAP, Supplemental Nutrition Assistance Program; HS, Head Start; WIC, Women, Infants, and Children.

* M_Diff_ = Estimated marginal group mean difference.

† se.

‡ Unadj. *P* = unadjusted *P* value.

§ B-H *P* = Benjamini–Hochberg adjusted *P* value.

|| *d* = Cohen’s *d*.

¶ B-H 95 % CI for *d* = Benjamini–Hochberg 95 % confidence interval for *d*.

## Discussion

Overall, households participating in both SNAP and HS were associated with the most favourable dietary environment among all program combinations. As indicated by the significant multivariate association across HDE indicators, SNAP + HS participants reported greater food and nutrition security, better control over dietary choices, perceived availability of healthy foods in nearby stores and fewer barriers to food access and utilisation, with small to moderate effect sizes (*d* = 0·2–0·5)^(15)^. The more favourable outcomes observed among SNAP + HS households may be due to several reinforcing mechanisms; however, this interpretation remains speculative, as the current data do not allow us to directly test these pathways. These findings nonetheless support the April 2022 policy change by the Administration for Children and Families and the Food and Nutrition Service on simplifying SNAP households’ access to HS, supporting the role of early childhood programs in fostering socio-economic stability and health^(28)^. First, HS’s participation in the Child and Adult Care Food Program may play a key role as all reimbursable meals served to HS-enrolled children can be claimed at the free rate, ensuring access to nutritious meals and alleviating household food burdens^(29)^. Second, HS’s unique federal performance standards, especially in nutrition and family engagement, can contribute to meeting children’s daily nutritional needs, fostering food security, and promoting healthy habits through family-style meals and nutrition education^(5,30,31)^. Third, HS exemplifies the successful implementation of and adherence to the Academy of Nutrition and Dietetics benchmarks through childcare providers’ healthful feeding practices, promoting healthy eating habits in young children^(32)^. Fourth, HS serves as an effective model, and if adopted by other childcare initiatives, such as the Child and Adult Care Food Program, it could yield significant benefits, improve dietary environments for young children more broadly and establish healthy weight trajectories in early childhood^(32)^. Furthermore, there is significant yet largely untapped potential in expanding Child and Adult Care Food Program eligibility to all children from SNAP-enrolled families in childcare, which could enhance access to essential nutrition and further reduce food insecurity among low-income households^(33)^. Finally, the integration of SNAP and HS simplifies administrative processes by eliminating duplicate income verification and fostering more efficient coordination across programs, ultimately improving accessibility and service delivery and hence improve outcomes across multiple HDE indicators^(34)^. This streamlined approach not only reduces administrative burdens and improves program accessibility but also aids in reaching families more effectively, creating a more stable and supportive dietary environment for young children to thrive.

SNAP + HS + WIC participants exhibited the lowest levels of household food security (i.e. the highest mean food insecurity scores) compared with other groups, suggesting that families with the greater socio-economic vulnerability may be more likely to participate in multiple assistance programs. In line with this, Gundersen *et al.* ^(35)^ reported that expanding programme participation to the most disadvantaged populations – those with the highest baseline food insecurity – can lower the average food security among participants. Specifically, they found that states offering expanded combined Temporary Assistance for Needy Families and SNAP benefits experienced wider gaps in food insecurity between participants and eligible non-participants.

Similarly, despite HS maintaining high-performance standards for nutrition and family engagement, the persistently low food security observed among SNAP + HS + WIC households likely reflects both their elevated baseline needs and the compounded challenges they face in managing multiple programmes. Already established FAP-related barriers, such as administrative and application burdens, limited programme or eligibility information^(36,37)^, can prevent income-eligible families living in under-resourced environments from fully benefiting. It is also plausible that self-selection bias and unobserved influencing factors like housing affordability or family support needs^(3)^ contribute to the lower food security observed in this group. Additionally, the variability in outcomes across different HDE indicators suggests that while participation in multiple programmes may improve certain aspects of HDEs – such as dietary choice or healthy food access – it does not necessarily translate into uniformly improved food security. These findings highlight the importance of further investigation into the specific structural and operational barriers that families face when attempting to coordinate and access multiple benefits. A better understanding of these challenges is essential to reducing enrollment burdens and enhancing program access and service utilisation.

Previous research has shown that households participating in both SNAP and WIC generally experience lower food insecurity compared with those enrolled in either programme alone^(8)^. Consistent with these findings, our unadjusted data show that SNAP + WIC households had lower mean food insecurity scores (8·41 (sd 4·35)) compared with SNAP-only households (8·99 (sd 4·52)), suggesting modest benefits from combined programme participation. However, after adjusting for relevant covariates, the food insecurity score for SNAP + WIC households was slightly higher than that of SNAP-only households. This discrepancy may be due to differences in sample composition, measurement sensitivity or unmeasured confounding factors – such as financial strain, housing instability or caregiving responsibilities^(3)^ – that influence food insecurity independent of programme participation.

Categorical thresholds based on the USDA’s Household Food Security Survey Module provide additional context. The raw mean scores of SNAP + HS households (M_unadj_ = 4·28) fell within the ‘low food security’ category, whereas SNAP-only (M_unadj_ = 8·99), SNAP + WIC (M_unadj_ = 8·41) and SNAP + WIC + HS (M_unadj_ = 9·83) households averaged scores within the ‘very low food security’ range. Even after adjusting for covariates, SNAP + HS households maintained significantly lower Household Food Security Survey Module marginal mean scores (M_adj_ = 6·23), indicating comparatively lower severity of food insecurity (Table 2). While these findings highlight the relative advantage of SNAP + HS participation, the fact that this group still falls within the ‘low food security’ category suggests substantial room for improvement. Strategies such as enhancing SNAP benefit adequacy and more fully leveraging HS’s family engagement and nutrition supports may further strengthen the HDE^(38,39)^.

Demographic differences between study groups also suggest variations in FAP outreach, cultural engagement and geographic access, necessitating additional research on how these factors influence equitable program access. Future studies should further examine how the impact of multiple FAP on HDE indicators varies by race, ethnicity and geography and explore opportunities to design culturally relevant interventions that are responsive to community contexts. Additionally, conducting targeted needs assessments among similar demographics and populations could help identify localised barriers and inform the development of inclusive program strategies.

### Strengths and limitations

The notable strengths of this study include a robust sample size and diverse demographics, offering a comprehensive representation of Nebraska’s low-income households, including those in rural areas and food deserts and across racial and ethnic groups. This enhances the generalisability of the findings within Nebraska’s low-income population. The methodological rigor of our statistical analysis, including controlling for confounding demographic variables and applying the Benjamini–Hochberg correction to control the false discovery rate, reduce our chances of finding spurious associations, further strengthens the validity of our results and ensures that our findings are robust and not driven by sample size. Furthermore, the recent Administration for Children and Families-Food and Nutrition Service policy changes, which streamline the process for SNAP families to access HS, amplify the relevance and applicability of our findings.

However, the cross-sectional design and lack of random assignment in study groups limit causal inferences. Reliance on self-reported data introduces potential response bias. Unavailability of data on how long households have been enrolled in the programmes and unaccounted confounding variables may affect the results. Future research should employ quasi-experimental or longitudinal designs to better clarify the directionality of relationships, assess the long-term impact of multiple program participation on HDE outcomes and mitigate the risk of reverse causality inherent in cross-sectional designs. The Nebraska-specific data may limit generalisability to other US states. Additionally, the study focused on combined participation in SNAP with WIC and/or HS, excluding households participating exclusively in both WIC and HS due to the relatively small sample size (*n* 56) below the a priori minimum sample size threshold. Future studies on these programmes could clarify their individual and joint effects. Another key limitation is the absence of child-level data on age. Since multiple children of varying ages from one household may participate in FAP, we did not control for individual child age, which may limit understanding of age-specific programme effects and HDE outcomes within multi-child households. Future research should collect detailed child-level enrollment and age data to better evaluate their effects on programme engagement and dietary environments.

### Conclusion

This exploratory study lays promising groundwork for future research, suggesting that combined participation in SNAP and HS can foster a more supportive HDE for young children in families by leveraging SNAP’s financial assistance for food and HS’s performance standards in nutrition and family engagement. This integration may lead to long-term health benefits by instilling healthy eating habits early in those children’s lives and reducing the risk of childhood obesity and diet-related chronic diseases in low-income households.

## Supporting information

Purkait et al. supplementary material Purkait et al. supplementary material

## Supplementary material

For supplementary material accompanying this paper visit https://doi.org/10.1017/S1368980025100864
