# Supplementary material for: Combined participation in the Supplemental Nutrition Assistance Program (SNAP) and Head Start is associated with healthy household dietary environments for young children in low-income families
Source: Public Health Nutr. 2025 Aug 22;28(1):e139. doi: 10.1017/S1368980025100864 (PMC12516603; doi:10.1017/S1368980025100864)
Supplement: Purkait et al. supplementary material [file S1368980025100864sup001.docx]

**Supplementary Material 1.** Survey Instruments used from the Healthy People, Healthy State

***(I) Food Security***

Please select the most relevant answer based on the following statements you have experienced about your family's food situation in the last 12 months.

A. We are worried whether our food would run out before we got moneyto buy more.

1. Often true
2. Sometimes true
3. Never true
4. Don't know

B. The food that we bought just didn’t last, and we didn’t have money toget more.

1. Often true
2. Sometimes true
3. Never true
4. Don't know

C. We couldn’t afford to eat balanced meals.

1. Often true
2. Sometimes true
3. Never true
4. Don't know

D. Did (you or other adults in your household) ever cut the size of yourmeals or skip meals because there wasn't enough money for food?

1. Yes
2. No
3. Don't know

E. How often did this happen?

1. Almost every month
2. Some months but not every month
3. Only 1 or 2 months
4. Don't know

F. Did you ever eat less than you felt you should because there wasn't enough money for food?

1. Yes
2. No
3. Don't know

G. Were you ever hungry but didn't eat because there wasn't enough money for food?

1. Yes
2. No
3. Don't know

H. Did you lose weight because there wasn't enough money for food?

1. Yes
2. No
3. Don't know

I. Did (you or other adults in your household) ever not eat for a whole day because there wasn't enough money for food?

1. Yes
2. No
3. Don't know

J. How often did this happen?

1. Almost every month
2. Some months but not every month
3. Only 1 or 2 months
4. Don't know

K. We relied on only a few kinds of low-cost food to feed our child(ren) because we were running out of money to buy food.

1. Often true
2. Sometimes true
3. Never true
4. Don't know

L. We couldn’t feed our child(ren) a balanced meal, because we couldn’t afford meals.

1. Often true
2. Sometimes true
3. Never true
4. Don't know

M. The child(ren) were not eating enough because we just couldn't afford enough food.

1. Often true
2. Sometimes true
3. Never true
4. Don't know

N. Did you ever cut the size of any of the child(ren)'s meals because there wasn't enough money for food?

1. Yes
2. No
3. Don't know

O. Did any of the child(ren) ever skip meals because there wasn't enough money for food?

1. Yes
2. No
3. Don't know

P. How often did this happen?

1. Almost every month
2. Some months but not every month
3. Only 1 or 2 months
4. Don't know

Q. Was your child(ren) ever hungry but you just couldn't afford more food?

1. Yes
2. No
3. Don't know

R. Did your child(ren) ever not eat for a whole day because there wasn't enough money for food?

1. Yes
2. No
3. Don't know

***(II) Nutrition Security***

A. In the last 12 months, we had to eat some foods that were not good for our health and well-being because we could not get other types of food.

1. Never
2. Rarely
3. Sometimes
4. Often
5. Always

B. In the last 12 months, we knew there were things we should or should not eat for our health and well-being but could not get healthful food.

1. Never
2. Rarely
3. Sometimes
4. Often
5. Always

C. In the last 12 months, we worried that the food we were able to eat would hurt our health and well-being.

1. Never
2. Rarely
3. Sometimes
4. Often
5. Always

D. In the last 12 months, we had to eat the same thing for several days in a row because we did not have money to buy other food.

1. Never
2. Rarely
3. Sometimes
4. Often
5. Always

***(III) Healthfulness Choice***

A. In the last 12 months, we could control if we were able to eat quality fruits and vegetables.

1. Never
2. Rarely
3. Sometimes
4. Often
5. Always

B. In the last 12 months, we could control if we were able to eat foods that were good for our health and well-being.

1. Never
2. Rarely
3. Sometimes
4. Often
5. Always

C. In the last 12 months, we could control if we did or did not have only processed foods from a box, bag, or can to eat (e.g., mac and cheese, ramen noodles, canned ravioli, frozen TV dinners, or other processed foods).

1. Never
2. Rarely
3. Sometimes
4. Often
5. Always

***(IV) Dietary Choice***

A. In the last 12 months, we had to eat some foods that we did not want to eat because we could not get other types of food.

1. Never
2. Rarely
3. Sometimes
4. Often
5. Always

B. In the last 12 months, the types of foods we ate were always changing because we did not know what we would be able to get to eat.

1. Never
2. Rarely
3. Sometimes
4. Often
5. Always

C. In the last 12 months, we had little control over the food we were able to eat.

1. Never
2. Rarely
3. Sometimes
4. Often
5. Always

***(V) Food Store Perceived Limited Availability***

A. In the last 12 months, the food stores we went to had very few quality fruits and vegetables.

1. Never True
2. Sometimes True
3. Often True

B. In the last 12 months, the food stores we went to had very few foods that we liked.

1. Never True
2. Sometimes True
3. Often True

C. In the last 12 months, we worried that the food we were able to eat would hurt our health and well-being.

1. Never True
2. Sometimes True
3. Often True

***(VI) Utilization Barriers***

A. In the last 12 months, we did not have access to a refrigerator, freezer, or other way to keep food from spoiling.

1. Never True
2. Sometimes True
3. Often True

B. In the last 12 months, we did not have a way to cook meals (e.g., stove, oven, microwave, hot plate or other appliance).

1. Never True
2. Sometimes True
3. Often True

C. In the last 12 months, we did not have the kitchen tools or utensils needed to cook meals (e.g., pots, pans, a stirrer, can opener, knife, spoons/forks, or other utensils).

1. Never True
2. Sometimes True
3. Often True

D. In the last 12 months, we did not have a clean and sanitary area to prepare meals.

1. Never True
2. Sometimes True
3. Often True

E. In the last 12 months, we did not know how to select healthy foods from the food options we had.

1. Never True
2. Sometimes True
3. Often True

F. In the last 12 months, we did not know how to make homemade meals from the food options we had (e.g., “meals from scratch” or meals without pre-made items).

1. Never True
2. Sometimes True
3. Often True

G. In the last 12 months, we could not make a healthy meal from the food options we had.

1. Never True
2. Sometimes True
3. Often True

H. In the last 12 months, we did not have time to cook meals.

1. Never True
2. Sometimes True
3. Often True

***(VII) Healthy Food Access Barriers***

**Please select the best option based on your household’s access to healthy foods for the following questions.**

How often do you face the following situations that make it hard to get healthy foods (healthy food includes fruits and vegetables, whole grains, beans and legumes, low-fat dairy, and lean meats)?

|  | Never | Rarely | Sometimes | Often | Always |
| --- | --- | --- | --- | --- | --- |
| A. The grocery store is far from my home |  |  |  |  |  |
| B. There are less transportation facilities near my home |  |  |  |  |  |
| C. The grocery store has limited hours |  |  |  |  |  |
| E. My family member(s) has physical disability |  |  |  |  |  |
| F. The food at my grocery store is not good quality |  |  |  |  |  |
| G. I do not have much time to go grocery shopping |  |  |  |  |  |
